# The Dual Nature of Sesamol: Tyrosinase-Dependent Bioactivation from an Inhibitor to a Cytotoxic Pro-Oxidant

**DOI:** 10.3390/ijms27198597

**Published:** 2026-09-25

**Authors:** Hitomi Tanaka, Tomoko Nishimaki-Mogami, Shosuke Ito, Kazumasa Wakamatsu

**Affiliations:** 1 Department of Medical Technology, School of Health Sciences, Gifu University of Medical Science, 795-1 Nagamine, Ichihiraga, Seki 501-3892, Gifu, Japan; hitanaka@u-gifu-ms.ac.jp; 2 Institute for Melanin Chemistry, Fujita Health University, 1-98 Dengakugakubo, Kutsukake-cho, Toyoake 470-1192, Aichi, Japan; sito@fujita-hu.ac.jp; 3 Division of Biochemistry, National Institute of Health Sciences, Kawasaki-ku, Kawasaki 210-9501, Kanagawa, Japan; mogami@nihs.go.jp

**Keywords:** anti-aging, melanin-producing cells, cytotoxic effects, pro-oxidant activity, quinone, sesamol, sesame

## Abstract

Sesamol (3,4-methylenedioxyphenol) is a unique natural phenolic compound derived from sesame seeds that is characterized by its concurrent phenolic and methylenedioxy moieties. The purpose of this study was to investigate the tyrosinase-mediated metabolic activation of sesamol to determine whether a cytotoxic quinone intermediate is produced, mirroring the mechanism established for *p*-substituted phenols such as resveratrol. Although sesamol is recognized as a potent tyrosinase inhibitor, we demonstrate that in the presence of catalytic amounts of L-DOPA, it serves as an efficient substrate for tyrosinase-mediated bioactivation. The resulting metabolite, sesamol-5,6-quinone, was identified via reduction with NaBH_4_ or ascorbic acid to its corresponding catechol, followed by isolation through acetylation to stabilize the transient species. The reactive nature of sesamol-5,6-quinone was confirmed through the formation of covalent adducts with *N*-acetylcysteine. In metabolic experiments using human tyrosinase-expressing 293T cells, we identified the reactive metabolite, sesamol-5,6-quinone, through the formation of intracellular thiol adducts released into the medium. While sesamol is systemically protective, it induced a marked tyrosinase-dependent cytotoxicity in B16BL6 melanoma cells, which was significantly suppressed by tyrosinase knockdown. Crucially, sesamol oligomers exhibited potent pro-oxidant activity, generating hydrogen peroxide and depleting reduced glutathione through its oxidation to GSSG. These findings suggest that tyrosinase-mediated conversion transforms sesamol into a highly reactive pro-oxidant, which induces substantial cytotoxicity in melanin-producing cells via protein arylation and profound redox imbalance.

## 1. Introduction

Sesamol (3,4-methylenedioxyphenol, (1)), a prominent natural phenolic lignan de-rived from sesame seeds (*Sesamum indicum*), possesses a unique molecular structure characterized by the co-existence of phenolic and methylenedioxy moieties [1] (Figure 1). Sesamol (1) is particularly abundant in roasted sesame oil—a dietary staple in East Asian cuisines (including Japan, Korea, and China)—where it is primarily formed through the thermal degradation of sesamolin (2) during high-temperature roasting and processing of sesame seeds (Figure 1). Historically, sesamol (1) has been celebrated for its potent antioxidant, anti-inflammatory, and anti-aging properties [2,3] and is recognized as a multi-targeted therapeutic agent with cardioprotective, neuroprotective, and hepatoprotective activities [1,4,5,6,7,8]. Furthermore, systematic reviews regarding the antioxidant and anti-inflammatory activities of sesame oil have been supported by chemical profiling and molecular docking studies [9]. The antioxidant capacity of sesamol (1) has been evaluated through electrochemical methods for radical scavenging activity, and various approaches have been proposed to develop more potent antioxidants, including computationally designed derivatives, chemical modifications at the C-6 position, and alkylated sesamol derivatives [10,11,12]. In the field of dermatology, sesamol (1) has conventionally been classified as a potent tyrosinase inhibitor and has been utilized for its anti-melanogenic properties [6]. Specifically, sesamol (1) has been shown to influence melanin synthesis through direct interaction with tyrosinase [1], and its efficacy in suppressing the generation of ultraviolet B (UVB)-induced reactive oxygen species (ROS) [13,14], as well as UV-induced hyperpigmentation and skin damage in mice, has been demonstrated. Moreover, the development and evaluation of sesamol (1) as a skin anti-aging agent is also underway. Sesamol (1) exhibits high oral bioavailability and can cross the blood–brain barrier, offering protection against oxidative stress-related pathologies such as Huntington’s and Parkinson’s diseases [15]. Recent comparative studies have revealed in detail the intracellular uptake pathways and cytotoxicity of sesamol (1) or sesamolin (2) in human melanoma cells (SK-MEL-2), noting that those compounds exhibit distinct cytotoxic potencies and vary in their rates of cellular accumulation [16,17]. However, the metabolic fate of sesamol (1) within melanocytes and its potential for pro-oxidant bioactivation have yet to be fully elucidated. Sesamol (1) has also been reported to inhibit melanogenesis by downregulating key enzymes and transcription factors, including tyrosinase, tyrosinase-related protein-1 (TRP-1), tyrosinase-related protein-2 (TRP-2), microphthalmia-associated transcription factor (MITF), and melanocortin 1 receptor (MC1R) [7,18,19].

The clinical significance of tyrosinase-mediated bioactivation was brought to the forefront by rhododendrol (4-(4-hydroxyphenyl)-2-butanol, RD, (3)), a potent leukoderma-induced skin-whitening agent. RD (3) serves as a substrate for tyrosinase, leading to the formation of reactive quinones and subsequent oxidative stress specifically in melanocytes [20]. This mechanism exemplifies the Systemic-Local Paradox, where a compound that is systemically safe or beneficial is ‘hijacked’ by tyrosinase to become a localized toxin. Whether sesamol (1) undergoes a similar toxicological transformation—acting as a suicide substrate—is yet to be fully elucidated. Such behavior, which distinguishes sesamol (1) from other sesame lignans like sesamolin (2), mirrors other *p*-substituted phenolic compounds, such as *trans*-resveratrol (RES, (4)), pterostilbene (PTS, (5)), and equol (EQ, (6)) [21,22,23] (Figure 2). Our previous investigations demonstrated that those molecules function as efficient substrates for tyrosinase, yielding highly reactive quinone intermediates that exert potent cytotoxicity against melanocytes via the arylation of cellular nucleophiles, such as protein thiols (Figure 2). Traditionally, in standard colorimetric assays, tyrosinase activity is conventionally quantified by measuring the absorbance of dopachrome—a red-to-orange pigmented metabolic intermediate in the melanin biosynthetic pathway with a characteristic absorption maximum at 475 nm. It is formed via the rapid intramolecular cyclization and subsequent oxidation of dopaquinone, which is produced when tyrosinase oxidizes L-tyrosine or L-DOPA. Dopachrome is eventually metabolized into the black pigment eumelanin, and its absorbance at 475 nm serves as a critical benchmark for measuring tyrosinase activity [24]. While sesamol (1) has been classified as a potent tyrosinase inhibitor (IC_50_ = 1.6 µM) due to its ability to suppress this 475 nm absorbance, our findings suggest that this low IC_50_ value does not reflect true enzyme inhibition but rather the high affinity of sesamol (1) as an efficient substrate that outcompetes L-DOPA for the tyrosinase active site. The reduction in 475 nm absorbance is thus a result of substrate competition, where the enzyme preferentially oxidizes sesamol (1) instead of L-DOPA. While sesamol (1) exhibits significantly more potent apparent inhibitory activity compared to common whitening agents like kojic acid and β-arbutin under these conditions, our study demonstrates a crucial function shift. When catalytic amounts of L-DOPA are present, sesamol (1) is bioactivated into the highly reactive and cytotoxic sesamol-5,6-quinone (7), effectively acting as an efficient substrate rather than a simple inhibitor (Figure 2). This sesamol-5,6-quinone (7), electrophilic species, binds to thiol groups (SH groups) of critical intracellular proteins and compounds. This arylation process disrupts cellular proteostasis and inactivates the functions of enzymes, leading to selective cytotoxicity in melanocytes [1,25,26]. Furthermore, the toxicity of sesamol (1) is amplified by its subsequent metabolites; sesamol-5,6-quinone (7) polymerizes into oligomers that act as potent pro-oxidants (Figure 2). These oligomers facilitate a robust redox cycle, generating hydrogen peroxide (H_2_O_2_) and depleting reduced glutathione (GSH) by oxidizing it to oxidized glutathione (GSSG). This sequence transforms a beneficial antioxidant into a deadly toxin specifically within tyrosinase-active environments, defining the ‘Systemic-Local Paradox.’ This term was popularized by social anthropologist Thomas Hylland Eriksen [27], who discussed it as a clash between the logic of global systems and local settings. Structurally analogous to his view, several studies in biochemistry have documented this phenomenon—the concept that an antioxidant that is systemically beneficial exhibits selective toxicity in an environment where a specific enzyme (tyrosinase) is present [26,27,28,29,30,31]. The shared structure of overall rationality leading to local breakdown across such disparate fields provides a profound framework for understanding cellular resilience through the lens of systems theory.

We employed chemical acetylation to stabilize and identify these metabolites. In the present study, we investigated the tyrosinase-dependent cytotoxicity of sesamol (1) in B16BL6 melanoma cells and validated its metabolic bioactivation using human tyrosinase-expressing 293T (hTYR-293T) cells under sub-cytotoxic conditions. We explored the pro-oxidant role of sesamol oligomers in generating H_2_O_2_ and causing the redox imbalance. The aim of this study was to provide a comprehensive mechanistic understanding of how the tyrosinase-mediated bioactivation of sesamol (1), involving the formation of reactive quinonoid intermediates and subsequent oxidative products into reactive catechols and oligomers, contributes to its selective toxicity toward melanin-producing cells.

## 2. Results

### 2.1. Tyrosinase-Catalyzed Oxidation of Sesamol (1) and Characterization of Products

Similar to RES (4) and PTS (5) [21,23], the tyrosinase-catalyzed oxidation of 100 µM sesamol (1) was initiated with 10 μM catalytic L-DOPA by mushroom tyrosinase (50 U/mL) in 50 mM sodium phosphate buffer at pH 6.8 at 37 °C. The UV/Visible spectral changes over 60 min showed the development of a sharp and high absorption band at 298 nm (sesamol (1) has an absorption maximum at 298 nm) during 10 min of the reaction (Figure 3a). In addition, the development of the 298 nm absorption was accompanied by the formation of a broad and weak absorption centered at approximately 400 nm, indicating the formation of a visible-absorbing oxidation product. To further characterize the reaction products, the reaction mixture was analyzed by reverse-phase C18 high-performance liquid chromatography (HPLC).

During this study, NaBH_4_ was used to reduce quinoid products to catechol structures to terminate the oxidation, as we have done in our previous studies [21,23,32,33]. However, we noticed that products after the NaBH_4_ reduction were not stable, and results of HPLC analysis became variable. Therefore, we chose to follow the oxidation after acidification by HClO_4_ without the reduction step. HPLC analysis showed three products, the major Compound **1** and minor Compounds **2** and **3**, when analyzed at 298 nm (Figure 3b). They had retention times (RT) of 5.9, 7.6, and 4.5 min (sesamol eluted at RT 22.4 min; Figure 3b), respectively. Compound **1** reached a maximum level at 15 min, when sesamol (1) had been completely consumed, whereas Compound **2** continued to increase throughout the reaction period. Compound **3** first became detectable at 15 min and gradually increased thereafter. Among these three products, Compounds **1** and **2** were also detected at 400 nm. On HPLC, Compounds **1** and **2** showed peak-height ratios at 298 and 400 nm (A298/A400) of 8.0 and 130, respectively. The ratio of 8.0 for Compound **1** was consistent with the A298/A400 ratio of 7.8 obtained from the UV/visible spectrum (Figure 3a). Based on these results, we tentatively assign that Compounds **1** and **2** correspond to sesamol-5,6-quinone (7) and sesamol-4,5-quinone (8), respectively (Table S1). These structure assignments are tentative (see below for the identification). Those *o*-quinones were relatively stable under the experimental conditions: Compound **1** gradually decreased by 12% during the 30–60 min incubation, whereas Compound **2** remained unchanged. Compound **3** was assumed to be a dimeric sesamol derivative based on its increase with time (Table S1).

The detection of relatively stable quinonoid molecules led us to examine the effect of ascorbic acid (AA). AA is known to have an ability to reduce many of *o*-quinones to the corresponding catechols [21,23,32,33]. UV-visible following of the tyrosinase-catalyzed reaction of 100 µM sesamol (1) in the presence of 1000 µM AA (10 molar eq.) was performed (Figure 4a). At 60 min, the absorbance at 400 nm in the presence of AA was reduced to approximately one-third of that observed in the absence of AA (Figure 4a). An absorption around 310 nm also developed during the reaction. These spectral changes suggest that AA alters the distribution of sesamol-derived oxidation products, consistent with the accumulation of reduced catecholic and secondary reaction products. In addition, the absorbance at 350 nm progressively increased over the 60 min reaction period (Figure 4a, inset). The accumulation of catecholic and oligomeric products was suggested.

HPLC following of the reaction yielded two new products Compounds **4** and **5** (RT of 11.7 and 5.5 min), respectively, in addition to Compounds **1**, **2**, and **3** (Figure 4b, Figure S1 for HPLC at 30 min), with Compound **4** being the major of the two newly formed products. As observed in the absence of AA (Figure 3c), Compounds **1** and **2** were also detected at 400 nm (Figure 4c). Compounds **4** and **5** were assumed to be catecholic, as they did not show absorption at 400 nm, and tentatively assigned the structures sesamol-5,6-catechol (9) and sesamol-4,5-catechol (10), respectively (Table S1). This progressive accumulation of Compound **3** was accompanied by the time-dependent increase in absorbance at 350 nm (Figure 4a, inset), providing additional spectroscopic support for the formation of a secondary coupling product. Among those products, only Compound **3** continued to increase as it did in the absence of AA (Figure 3b). On the other hand, the other products (Compounds **1**, **2**, **4**, and **5**) were rather unstable; they decreased during the 30–60 min reaction time by 35%, 20%, 77%, and 9%, respectively. The assignment of Compound **4** as sesamol-5,6-catechol (9) is consistent with its rapid disappearance, as sesamol-5,6-catechol (9) is likely more reactive than sesamol-4,5-catechol (10) due to the steric hindrance of the latter. It appears likely that Compound **1** (electrophilic *o*-quinone) and Compound **4** (nucleophilic catechol) reacted to form a dimeric adduct, Compound **3**. It should be noted that Compound **3** was produced 13-fold more in the presence of AA compared to its absence, indicating the accelerating effect of catecholic monomer(s) in the production of a dimeric Compound **3**.

To establish chemical structures of these highly unstable catechols (Compounds **4** and **5**), rapid extraction with ethyl acetate followed by acetylation was performed. Compound **4** (9) was identified as 5,6-dihydroxy-1,3-benzodioxole (sesamol-5,6-catechol (9), Table S1). ^1^H-NMR analysis (CD_3_OD) of its stable diacetyl derivative (5,6-diacetoxy-1,3-benzodioxole, (11)) showed a distinct singlet at δ 6.72 ppm (integrating for two protons, C4-H and C7-H), proving a highly symmetrical molecular structure and confirming that tyrosinase regioselectively hydroxylates sesamol (1) at the vacant C6 position, which is *para* to the methylenedioxy group (Figure S2, Tables S1 and S2). Compound **2** (8) was predicted to be a positional isomer of compound **1** (7) (Table S1). Unfortunately, due to its extremely low yield and high redox instability under tyrosinase-catalyzed oxidation, direct preparative isolation of compound **2** (8) from the enzymatic reaction mixture was not feasible. To verify its structural identity, a non-enzymatic chemical oxidation method (modeled after the chemical oxidation of phenols to catechols by Manini et al. [26]) was employed to oxidize sesamol (1). This chemical approach successfully yielded the isomeric catechol, which was subsequently trapped and stabilized as 4,5-diacetoxy-1,3-benzodioxole (12). ^1^H-NMR analysis (CDCl_3_) of this acetylated isomer exhibited a complete loss of symmetry, resolving into two distinct aromatic doublets at δ 6.65 ppm (d, 1H, J = 6.0 Hz) and 6.67 ppm (d, 1H, J = 6.0 Hz), unequivocally establishing the 4,5-substituted structure (Figure S3, Tables S1 and S2).

### 2.2. Reaction of Sesamol-5,6-Quinone (7) with Non-Protein Thiol Compounds Producing Mono-Adduct

Sesamol-5,6-quinone (7) was investigated for its ability to bind to thiol compounds, using *N*-acetyl-L-cysteine (NAC) as a model for biologically important thiols. The 100 µM sesamol (1) was oxidized with tyrosinase (50 U/mL) in the presence of 400 µM (4 molar excess) NAC and 10 µM L-dopa. Surprisingly, UV-visible spectral following of the reaction (Figure 5a) gave changes similar to those in the absence of NAC (Figure 3a), suggesting that sesamol-5,6-quinone (7) (and sesamol-4,5-quinone (8)) are rather resistant to nucleophilic addition of NAC.

HPLC analysis of the reaction course showed that the quinonoid Compounds **1** and **2** were indeed the major products (Figure 5b). However, Compound **6** was newly detected, although at low levels. A catecholic nature of Compound **6** was confirmed by the absence of absorption at 400 nm (Figure 5c).

To isolate the NAC adduct, a tyrosinase-catalyzed oxidation was performed on a preparative scale using 0.9 mM sesamol (1), 0.1 mM L-DOPA, and 4 mM NAC for 30 min. Upon reduction with NaBH_4_, preparative HPLC [32] was performed to isolate a major product, which was identified as a mono-NAC derivative of sesamol catechol (4-NAC-5,6-dihydroxy-1,3-benzodioxole (13)) through ^1^H-NMR and ^13^C-NMR analysis (Figures S4 and S5, Tables S1 and S2).

The structural assignment of Compound **6** as 4-NAC-5,6-dihydroxy-1,3-benzodioxole (13) led us to assign Compound **1** as 1,3-benzodioxole-5,6-quinone (7). Likewise, Compound **2** can be assigned the structure 1,3-benzodioxole-4,5-quinone (8) (Table S1).

### 2.3. Metabolism of Sesamol (1) in Human Tyrosinase-Expressing 293T (hTYR-293T) Cells

To verify whether sesamol (1) is effectively oxidized to its quinone derivative (7) and subsequently conjugates with intracellular thiols in vitro, we utilized an hTYR-293T cell model. This experimental system was selected based on our established findings that various phenols linked to leukoderma undergo efficient metabolic activation to quinones within these cells [34]. To assess metabolic pathways independently of direct cellular damage, hTYR-293T cells were treated with a sub-cytotoxic concentration of sesamol (1) (1.0 mM) for a duration of 2 h (Figure S6a).

The functional activity of tyrosinase in the transfected cells was confirmed by monitoring the response to 4-*S*-cysteaminylphenol (4SCAP (14), 0.3 mM), a selective anti-melanoma agent (Figure S6b). As previously documented [34], 4SCAP (14) triggers cell death in a tyrosinase-dependent manner, significantly reducing the viability of both hTYR-293T and B16BL6 melanoma cells.

Sesamol (1) was taken up by cells in proportion to the exposure dose, but no adducts were detected in the cells (Figure 6a). Since the amount of sesamol (1) taken up by the cells was at a lower concentration than in the medium, it is assumed that even if adducts were produced, they were below the detection limit. In the culture medium, thiol adducts were detected in a dose-dependent manner following the addition of sesamol (1). Given that these metabolites were not detected within the cells, these results suggest their rapid excretion from the cells into the medium. As shown in Figure 6b, sesamol (1) was taken up by hTYR-293T cells and was metabolized to cysteine (CySH) adduct and GSH adduct of sesamol-5,6-quinone (7), respectively, in a dose-dependent manner. Ten min after initiating the tyrosinase-catalyzed oxidation of sesamol (1) in the presence of either GSH or CySH (at which point the starting material, sesamol (1), had completely disappeared), the newly formed adducts were analyzed by HPLC. These reaction products served as standards to quantify the total amount of thiol adducts produced intracellularly and released into the culture medium. Despite testing various HPLC conditions to separate the catechol adducts of CySH and GSH, they co-eluted at a retention time of 11.8 min. Therefore, following established methods for RES (4) and PTS (5) [21,33], the quantities are expressed as peak heights relative to the initial peak height of 100 µM sesamol (1). As a result, we found that adducts were released into the medium in a manner dependent on the amount of sesamol (1) added.

### 2.4. Tyrosinase-Dependent Cytotoxicity in B16BL6 Melanoma Cells

Next, we investigated the effect of tyrosinase on tyrosinase-dependent cytotoxicity by knocking down tyrosinase with siRNA. In B16BL6 melanoma cells, transfection with tyrosinase-targeting siRNA for 24 h effectively reduced tyrosinase mRNA expression levels (Figure 7a). The cells were exposed to the indicated concentrations of 4SCAP (14) or sesamol (1) for 24 h, after which the viability was measured. Upon exposure to 4SCAP (14), tyrosinase-dependent enhanced cytotoxicity was observed (Figure 7b). Tyrosinase knockdown inhibited the cytotoxicity induced by sesamol (1) treatment for 24 h in a dose-dependent manner compared with B16BL6 melanoma cells transfected with a negative control siRNA (Figure 7c). The *p*-values of siRNA Ctrl and siRNA Tyr at 100 µM sesamol were *p* < 0.05. As a result, sesamol (1) significantly contributed to tyrosinase-dependent cytotoxicity.

### 2.5. Pro-Oxidant Activity of Sesamol Oligomers

Building on evidence that tyrosinase-derived oligomers of phenols like RD (3), RES (4), and EQ (6) act as pro-oxidants, we examined the pro-oxidant potential of sesamol (1)-derived oligomers [20,21]. These oligomeric species were synthesized by exposing 1 mM sesamol (1) to tyrosinase (100 U/mL) for 120 min at pH 7.4. Following their formation, the oligomers were reacted with 1 molar equivalent of GSH. The depletion of GSH and the subsequent accumulation of GSSG were monitored for 60 min using a specialized HPLC protocol with 3,5-di-*tert*-butyl-1,2-benzoquinone (DBBQ) derivatization [33,35] (Figure 8a). Additionally, we measured the concomitant generation of H_2_O_2_. As depicted in Figure 8a,b, incubation with the oligomers led to a 60% reduction in GSH levels, partly due to its conversion into GSSG. This oxidative process was accompanied by the production of 25 µM H_2_O_2_, a significant increase over the 16 µM detected in the control group.

## 3. Discussion

Our findings reveal the ‘dual nature’ of sesamol (1) as it undergoes the tyrosinase-mediated bioactivation into cytotoxic pro-oxidants. While sesamol (1) exhibits a characteristic lag time in monophenolase activity [1], the addition of catalytic amounts of L-DOPA initiates the catalytic cycle by reducing the enzyme’s active site copper to the *deoxy*-tyrosinase state. Mechanistically, sesamol (1) possesses an extremely high affinity for the tyrosinase active site. In the absence of catechol substrates, sesamol (1) binds to *met*-tyrosinase, effectively acting as a potent inhibitor (IC_50_ = 1.6 µM) [6]. However, in our system, substrate competition occurs where tyrosinase preferentially oxidizes sesamol (1) into sesamol-5,6-quinone (7). This transition from an inhibitor to an efficient substrate mirrors the behavior of other *p*-substituted phenols, most notably the leukoderma-inducing agent RD (3), as well as RES (4), PTS (5), and EQ (6) [21,23,33]. This pseudo-inhibitory effect of sesamol (1) on dopachrome formation can be explained by substrate competition at the tyrosinase active site. When L-DOPA and sesamol (1) are concurrently present, tyrosinase preferentially oxidizes sesamol (1) to its reactive quinones (7,8) rather than converting L-DOPA into dopachrome. Consequently, the decrease in the absorbance at 475 nm (the peak wavelength for dopachrome) does not indicate the blockade of the enzyme but rather highlights that sesamol (1) acts as an efficient alternative substrate that undergoes metabolic bioactivation, driving the ‘Systemic-Local Paradox’ within active melanocytes. Interestingly, other sesame lignans such as sesamolin (2) have been shown to prevent oxidative damage by activating the Nrf2-ARE signaling pathway [4,16]. While sesamol (1) likely shares these protective properties, the presence of tyrosinase creates a metabolic shortcut that overrides these defensive mechanisms. In tyrosinase-active cells, the rapid generation of sesamol-5,6-quinone (7) and subsequent GSH depletion occur too quickly for the Nrf2-mediated antioxidant response to compensate, leading to lethal oxidative stress.

While quinones are generally characterized as highly reactive electrophiles, their chemical stability varies significantly depending on their molecular structure. The common perception of quinones as ‘highly transient’ or ‘unstable’ species primarily applies to those capable of rapid intramolecular cyclization, such as dopaquinone and dopamine-quinone, which possess side-chain amino groups. In contrast, as discussed in our previous review [36], sesamol-5,6-quinone (7) lacks such a nucleophilic side chain for cyclization. This structural feature allows it to remain relatively stable in the quinoid state, facilitating its capture by external nucleophiles or its reduction back to the catechol form. The oxygen atoms of the methylenedioxy moiety, located at the *meta*-position relative to the *o*-quinone carbonyls, exert a powerful cooperative resonance electron-donating effect. This ‘push-pull’ electronic interaction between the electron-donating 1,3-dioxole ring and the electron-withdrawing *o*-quinone chromophore stabilizes the excited state and mesomeric zwitterionic resonance structures of sesamol-5,6-quinone (7), creating a thermodynamic energy well that confers remarkable chemical stability upon the monomeric quinone and delays its irreversible breakdown.

Specifically, our results support the regioselective formation of sesamol-5,6-quinone (7) during tyrosinase-mediated oxidation of sesamol (1). The regiochemistry of this pathway was established by reductive trapping of the oxidized intermediate as 5,6-dihydroxy-1,3-benzodioxole (9), followed by acetylation and ^1^H-NMR characterization of its stable diacetyl derivative, 5,6-diacetoxy-1,3-benzodioxole (11). This regioselectivity is consistent with the kinetic study by Fenoll et al. [37] on 3-hydroxyanisole (3-methoxyphenol), which reported that tyrosinase-catalyzed hydroxylation occurs preferentially at the position *para* to the methoxy substituent, which corresponds to the C6 position in sesamol (1).

A surprising finding in our in vitro spectral monitoring (Figure 5a) was that when sesamol (1) was oxidized by tyrosinase in the presence of 4 molar equivalents of NAC, the UV-visible absorption spectra were nearly identical to those recorded in the absence of NAC (Figure 3a). This indicates that under dilute, in vitro analytical conditions, sesamol-5,6-quinone (7) exhibits a surprising resistance to nucleophilic addition by NAC. This kinetic resistance is a direct consequence of the zwitterionic resonance stabilization described above, which reduces the electrophilicity of the C4/C7 positions relative to un-stabilized *o*-quinones. However, this in vitro resistance does not imply inertness in a biological environment.

This study indicated a remarkable stability of sesamol-5,6-quinone (7), the major product of tyrosinase-catalyzed oxidation of sesamol (1). This can be ascribed to the electron-donating ability of the methylenedioxy moiety, located at *meta*-position to the *o*-quinone moiety. However, this property of sesamol-5,6-quinone (7) does not necessarily mean the stability in biological environment. In this study, it was revealed that the presence of AA remarkably affects the metabolic course of sesamol-5,6-quinone (7), mimicking in vivo situation. Likewise, the addition reaction of NAC toward sesamol-5,6-quinone (7) would be accelerated under higher pH conditions at higher concentrations (e.g., GSH is present at millimolar concentrations).

Previous studies have reported that sesamol (1) inhibits melanogenesis by downregulating key transcription factors and enzymes, such as MITF, TRP-1, TRP-2, and MC1R [7,18,19]. Our findings suggest that the impact of sesamol (1) on melanocytes is multi-faceted: while it may reduce melanin synthesis through transcriptional regulation at lower concentrations, it simultaneously poses a risk of direct cytotoxicity via bioactivation when tyrosinase is enzymatically active. The final biological outcome—protection versus toxicity—may depend on the balance between its beneficial antioxidant effects and the rate of its metabolic conversion into reactive quinones. The biological consequences of this metabolic pathway were validated using two complementary cell models. In B16BL6 melanoma cells, which possess high levels of endogenous tyrosinase, sesamol (1) induced a marked, dose-dependent cytotoxicity that was significantly rescued by siRNA-mediated tyrosinase knockdown. This demonstrates that tyrosinase acts as the ‘executioner’ enzyme required to trigger the lethality of sesamol (1). Furthermore, hTYR-293T cells provided a specialized model for metabolic validation under sub-cytotoxic conditions, confirming that the intracellular generation and subsequent excretion of thiol adducts is directly linked to tyrosinase activity. The evaluation of tyrosinase-dependent metabolic activation and subsequent cytotoxicity using primary normal human melanocytes is scientifically challenging due to extreme donor-to-donor variability in their cellular responses. For instance, human melanocytes from different origins exhibit a wide range of sensitivities to the reference leukoderma-inducing agent RD (3), with reported IC_50_ values varying widely from micromolar to millimolar levels. To bypass this donor-dependent inconsistency and ensure high reproducibility, we established and utilized two complementary, well-controlled cell models. First, we employed hTYR-293T cells, prepared by transient transfection of a human tyrosinase expression plasmid into 293T cells obtained from the American Type Culture Collection (ATCC) (as described in Section 4.5 and Section 4.8), to perform precise, high-yield metabolite analyses under sub-cytotoxic conditions. Second, we utilized B16BL6 mouse melanoma cells to verify tyrosinase-dependent cytotoxicity, as they highly express endogenous tyrosinase which can be selectively knocked down using siRNA transfection to serve as a robust negative control. Regarding the study duration, while long-term safety evaluations are crucial for translational human applications, significant tyrosinase-dependent cytotoxicity was clearly detected in B16BL6 cells following only a 24 h exposure to sesamol (1), demonstrating that the metabolic bioactivation cascade and subsequent cell death proceed rapidly within active melanogenic cells.

Next, our analysis further demonstrated that the cytotoxicity of sesamol (1) is driven by two synergistic mechanisms (direct damage via thiol arylation and indirect damage via pro-oxidant activity).

(1) Direct Damage (Thiol Arylation): The electrophilic quinones covalently bind to intracellular protein thiol groups (cysteine residues) and small-molecule thiols such as GSH and CySH. This arylation disrupts cellular proteostasis and inactivates critical enzymes, leading to direct functional destruction of the cell.
(2) Indirect Damage (Pro-oxidant Activity): the quinone polymerizes into oligomers that facilitate a robust redox cycle that generates H_2_O_2_ and depletes reduced GSH by oxidizing it to GSSG, thereby driving the cell into a state of lethal oxidative stress. These oligomers act as potent pro-oxidants, generating 25 µM H_2_O_2_ and inducing a 60% depletion of reduced GSH through its oxidation to GSSG.

Our comparative potency analysis (Table 1) reveals a clear hierarchy of the pro-oxidant activities of these *p*-substituted phenolic oligomers: RD (3) > sesamol (1) > EQ (6) > RES (4)/PTS (5). Within this context, it is noteworthy that sesamol (1) exhibits a high potency second only to RD (3), a well-known leukoderma-inducing *p*-substituted phenol. Notably, while RES (4) and PTS (5) oligomers produce negligible H_2_O_2_, sesamol (1) generates levels exceeding those of EQ (6), making it a significantly more potent pro-oxidant. These findings define the ‘Systemic-Local Paradox.’ Several papers have been published regarding this Systemic-Local Paradox—the concept that an antioxidant which is beneficial systemically exhibits selective toxicity in an environment where a specific enzyme (tyrosinase) is present [26,28,29,30,31]. Sesamol (1) provides systemic protection in most tissues that lack tyrosinase, such as the brain and heart, by acting as a radical scavenger [38,39,40]. However, within tyrosinase-active melanocytes, the molecule is ‘hijacked’ by the enzyme and converted into a targeted toxin. This selective toxicity necessitates a careful re-evaluation of sesamol (1) and related phenolics in cosmetic and skin-whitening applications, as their safety profile is entirely dependent on the specific metabolic landscape of the target cells. While sesamol (1) is a common dietary component derived from roasted sesame oil and exhibits high oral bioavailability, our results highlight a critical distinction between systemic ingestion and localized skin application. In systemic circulation, sesamol (1) acts as a multi-organ protector. However, the topical application of sesamol (1) in high concentrations on melanocyte-rich areas could bypass systemic detoxification and directly trigger the tyrosinase-mediated pro-oxidant cascade, potentially leading to melanocyte damage similar to other leukoderma-inducing agents.

Ultimately, our data suggest the existence of a ‘metabolic threshold’ for sesamol (1). Below that threshold, its radical-scavenging activity may predominate, but exceeding it allows for the accumulation of reactive oligomers and H_2_O_2_ that overwhelm cellular defenses. Defining this threshold in human skin models will be essential for the safe pharmaceutical and nutraceutical application of sesamol (1), ensuring that its benefits as an antioxidant are not subverted by its potential as a pro-oxidant.

## 4. Materials and Methods

### 4.1. Materials

Sesamol (1) was purchased from Tokyo Chemical Industries (Tokyo, Japan). Tyrosinase (from mushrooms, specific activity 8503 U/mg), horseradish peroxidase, Ampliflu^TM^ Red reagent (10-acetyl-3,7-dihydroxyphenoxazine), L-cysteine (CySH), reduced glutathione (GSH), oxidized glutathione (GSSG), *N*-Acetyl-L-cysteine (NAC), 3,4-dihydroxyphenyl-L-alanine (L-DOPA), and Dulbecco’s Modified Eagle’s Medium (DMEM) containing 10% fetal bovine serum were purchased from Sigma-Aldrich (St. Louis, MO, USA). Perchloric acid (HClO_4_) was purchased from Katayama Chemical Industries Co., Ltd. (Osaka, Japan). Formic acid, NaBH_4_, ethyl acetate, and methanol (HPLC grade) were from FUJIFILM Wako Pure Chemical Corporation (Osaka, Japan). The highest purity Milli-Q water (Milli-Q Advantage, Merck Millipore Co., Tokyo, Japan) was used throughout this study to avoid contamination with metal ions.

### 4.2. Instruments

An HPLC system was used to follow the course of tyrosinase oxidation. It comprised an analytical a JASCO UV/VIS detector, a JASCO pump (JASCO Co., Tokyo, Japan), and a C18 column (Capcell Pak MG; 4.6 × 250 mm; 5 µm particle size, Osaka Soda, Osaka, Japan). The mobile phase was 0.4 M formic acid: methanol, 30:70 (*v*/*v*), 70:30 (*v*/*v*) or 95:5 (*v*/*v*). Analyses were performed at 45 °C or 40 °C at a flow rate of 0.7 mL/min or 1.0 mL/min. For preparative separation, a C18 preparative column (Capcell Pak MG; 20 × 250 mm; 5 µm particle size, Osaka Soda, Osaka, Japan) was used at a flow rate of 7.0 mL/min with the mobile phase (0.4 M formic acid: methanol, 30:70 (*v*/*v*)) at 40 °C. A Shiseido electrochemical detector (Shiseido, Tokyo, Japan) was also used to analyze low concentrations of metabolites, as electrochemical detection is >10 times more sensitive than UV/visible detection.

UV-visible spectra were measured with a JASCO V-630 UV-VIS spectrophotometer (JASCO Co., Tokyo, Japan). For measurements of H_2_O_2_, the maximum absorption wavelength of 568 nm possessed by red fluorescent resorufin in which Ampliflu^TM^ Red was oxidized was used.

Nuclear magnetic resonance (NMR, 400 MHz for ^1^H) spectra were obtained using a JNM-ECZ400S spectrometer (JEOL Ltd., Tokyo, Japan). ^1^H-NMR (400 MHz) and ^13^C-NMR (100 MHz) spectra were obtained in CD_3_OD and CDCl_3_. Chemical shifts are shown in ppm relative to the solvent signal at 3.30 ppm (CD_3_OD) or 7.26 ppm (CDCl_3_) in ^1^H-NMR, and 77.0 ppm in ^13^C-NMR, respectively.

### 4.3. Oxidation of Sesamol (1) by Tyrosinase in the Absence or Presence of AA or NAC

Following the method described by Ito et al. [33], a solution (2 mL) of 100 µM sesamol (1) and 10 µM L-DOPA was oxidized by 50 U/mL tyrosinase at 37 °C in 50 mM sodium phosphate buffer (pH 6.8). Changes in the absorption spectrum were followed for up to 60 min. The oxidation was also carried out in the presence of 1 mM AA or 0.4 mM NAC. For spectrophotometric analysis, the reference cell contained the same concentrations of buffer, tyrosinase, and AA or a thiol. For HPLC analysis, the reaction was stopped by adding a 200 µL aliquot to 200 µL 0.8 M HClO_4_.

### 4.4. Isolation of Sesamol Catechol Derivatives

According to the method described by Ito et al. [33], a solution of 0.1 mmol sesamol (1) (13.8 mg) in ethanol (2 mL) and 0.01 mmol L-DOPA (1.97 mg) in 200 µL 0.1 M HCl was mixed with 97.8 mL 50 mM sodium phosphate buffer (pH 6.8). The mixture was vigorously shaken at 37 °C, to which tyrosinase (20,000 U) in 1 mL buffer was added. After 5 min of oxidation, 2 mL 6 M HCl was added to stop the oxidation, and the mixture was extracted twice with 100 mL ethyl acetate. The reaction mixture was acetylated with 1 mL acetic anhydride and 1 mL pyridine. Residual pyridine was removed by azeotroping with 1 mL toluene. The obtained reaction mixture was separated by silica gel chromatography; following thin-layer chromatography and HPLC analysis, the main product was identified as the diacetyl derivative of sesamol catechol (5,6-diacetoxy-1,3-benzodioxole (11)) through NMR structural analysis. ^1^H-NMR of 5,6-diacetoxy-1,3-benzodioxole (11) (CD_3_OD): 2.21 (s, 6H), 5.99 (s, 2H), 6.72 (s, 2H) (Figure S2 and Table S2). UV (in 50 mM sodium phosphate buffer (pH 6.8) containing 1% ethanol): λ_max_ 288 nm (ε = 4.5 ×10^3^) and 232 nm (ε = 4.4 × 10^3^).

Because compound **2** (8) is generated in very low yields under tyrosinase-catalyzed oxidation and rapidly undergoes redox-driven oligomerization, its direct preparative isolation from the enzymatic reaction mixture was unsuccessful. To establish its chemical identity, we synthesized the authentic compound using a modified non-enzymatic chemical oxidation method by Manini et al. [26]. Briefly, sesamol (1) (27.6 mg, 0.2 mmol) was dissolved in 50 mL of chloroform/methanol mixture (9:1, *v*/*v*) and oxidized using *o*-iodoxybenzoic acid (IBX, 112 mg, 0.40 mmol) at room temperature for 30 min to selectively convert the monophenol to the corresponding catechols. One hundred mL of 100 mM phosphate buffer (pH 7.4) was added to the reaction mixture and was extracted twice with 50 mL chloroform. The combined chloroform extracts were dried over approximately 30 g of anhydrous sodium sulfate and filtered. The filtrate was concentrated under reduced pressure. The resulting residue was dissolved in 2 mL of ethyl acetate, and 100 mL of 100 mM phosphate buffer (pH 7.4) containing sodium dithionite (0.8 mmol, 4 equiv.) was added. The mixture was vigorously mixed at room temperature for 1 h to reduce the oxidation products. The reaction mixture was then extracted twice with 100 mL of ethyl acetate. The upper organic layers were collected and concentrated under reduced pressure. The crude, highly unstable catechol mixture was immediately acetylated by treatment with acetic anhydride (1 mL) and pyridine (1 mL) at room temperature overnight. After evaporation of volatiles, residual pyridine was removed by azeotroping with toluene (1 mL). The residue was purified by silica gel column chromatography (hexane/ethyl acetate, 8:2, *v*/*v*) to yield 4,5-diacetoxy-1,3-benzodioxole (12) (4.1 mg, 17% yield). ^1^H-NMR of 4,5-diacetoxy-1,3-benzodioxole (12) (CDCl_3_): 2.03 (s, 3H), 2.26 (s, 3H), 6.00 (d, 2H, J = 7.6 Hz), 6.65 (d, 1H, J = 6.0 Hz), 6.67 (d, 1H, J = 6.0 Hz) (Figure S3 and Table S2).

Regarding the synthesis of NAC-derivatives of sesamol (1), 0.9 mM sesamol (1), 0.1 mM L-DOPA, and 4 mM NAC (in 100 mL of 50 mM sodium phosphate buffer (pH 6.8) containing 2% ethanol) were oxidized using tyrosinase (20,000 U/mL) for 30 min. Two products were eluted at retention times of 5.8 min and 7.8 min at analytical HPLC (conditions: 40 °C, flow rate 0.7 mL/min, 0.4 M HCOOH:MeOH = 80:20). For the isolation of NAC adducts via preparative HPLC, the reaction mixture was first treated with NaBH_4_ to reduce the stable quinone adducts into their corresponding catechol forms. This reduction step was necessary to ensure efficient separation and structural stabilization during the collection of the adducts. One of these two compounds (a retention time of 5.8 min on analytical HPLC) was isolated by preparative HPLC and was identified as 4-NAC-5,6-dihydroxy-1,3-benzodioxole (13). ^1^H-NMR (CDCl_3_): 2.14 (s, 3H), 2.99 (1H, m), 3.10 (1H, m), 4.89 (1H, m), 5.89 (s, 2H), 6.41 (1H, s). ^13^C-NMR: 23.0, 27.0, 53.5, 98.2, 101.2, 108.1, 140.5, 141.5, 148.5, 150.5, 171.0, 172.5 (Figure S4 and Table S2).

### 4.5. Metabolism of Sesamol (1) in hTYR-293T Cells

The human tyrosinase-expressing hTYR-293T cells used in this study were prepared by transient transfection of a human tyrosinase expression plasmid into 293T cells obtained from the American Type Culture Collection (ATCC). Detailed preparation protocols are described below. Briefly, the human tyrosinase gene was transiently transfected into hTYR-293T cells (0.75 × 10^6^ cells) using Lipofectamine 2000 reagent (Invitrogen, Carlsbad, CA, USA) with the expression plasmid pcDNA3.1 containing the coding sequence of the human TYR gene, according to the method we previously reported [34]. Next, cells were cultured in DMEM containing 10% fetal bovine serum, and after 24 h, the medium (0.75 mL) was replaced with medium containing 0.0, 0.05, 0.1, or 0.2 mM sesamol. The incubation was then continued for 2 h. Medium samples (0.18 mL) were deproteinized with 4 M HClO_4_ at 4 °C for 60 min. These cell samples (0.75 × 10^6^ cells) were extracted by vigorously shaking with 0.4 M HClO_4_ for 60 min. After centrifugation at 10,000× *g* for 3 min, the supernatant was analyzed by HPLC as described below.

### 4.6. Adduct Formation of Sesamol-5,6-Quinones (7) with CySH and GSH

The 100 µM sesamol (1) was oxidized with tyrosinase (50 U/mL) in the presence of 3 mol eq. CySH or GSH at pH 6.8. In the HPLC analysis using a specific mobile phase (0.4 M HCOOH:MeOH = 95:5, at 45 °C), the retention time for both adducts was 11.8 min. Due to co-elution under these specific mobile phase conditions, the total amount was calculated as a combined value. This methodology was employed to ensure high-sensitivity detection of the total thiol adducts released, following established protocols for RES (4) and PTS (5) where peak heights were expressed relative to the initial signal of 100 μM sesamol (1).

### 4.7. Evaluation of Tyrosinase-Dependent Cytotoxicity in B16BL6 Melanoma Cells

We obtained B16BL6 mouse melanoma cells from the RIKEN BioResource Center (Tsukuba City, Ibaraki Prefecture) and cultured them in RPMI1640 medium containing 10% fetal bovine serum (FBS). The tyrosinase-dependent cytotoxicity of sesamol (1) was evaluated using the method we previously reported [34]. For siRNA knockdown of tyrosinase expression, cells (1 × 10^4^ cells/well) were transfected with 1 pmol Stealth™ Select RNAi (Thermo Fisher Scientific, Waltham, MA, USA) for mouse Tyr (MSS212191) or Stealth RNAi^TM^ negative control using Lipofectamine RNAiMAX reagent (0.2 µL/well) (Thermo Fisher Scientific). Cells were cultured for 24 h after transfection, and the culture medium was replaced with growth medium (100 µL) containing solvent (DMSO, 0.5%). Sesamol (1) at each concentration was added at this time. Cell viability was measured using the CellTiter-Glo^®^ luminescent cell viability assay (Promega Corp., Madison, WI, USA). To confirm whether tyrosinase knockdown had occurred, the expression level of Tyr mRNA in cells was measured 24 h after transfection and again 24 h after culturing in growth medium. Total RNA was extracted using a RNeasy Mini Kit with deoxyribonuclease (QIAGEN, Valencia, CA, USA). Quantitative real-time RT-PCR was performed using the QuantiTect Probe RT-PCR (QIAGEN), and the detection system used was the ABI Prism 7300 sequencing detection system (Thermo Fisher Scientific). Using primer/probe sequences of mouse Tyr (forward: 5′-AGCCTGTGCCTCCTCTAAGAACT-3′, reverse: 5′-CTTGCCGATGGCCAGAAG-3′; probe: 5′-6-FAM-TTGCAAAA/ZEN/GAATGCTGCCCACCA-IBFQ-3′) (Integrated DNA Technologies, Coralville, IA, USA) and 18S rRNA (Applied Biosystems, Thermo Fisher Scientific), expression data were normalized at the 18S rRNA level and are reported as a fold difference between control cells and Tyr-siRNA-treated cells.

### 4.8. Evaluation of Tyrosinase-Dependent Cytotoxicity in hTYR-293T Cells

To evaluate tyrosinase-dependent cytotoxicity, hTYR-293T cells (3 × 10^4^ cells/well) were transfected into 96-well plates with either a human tyrosinase expression plasmid (80 ng) or an empty vector (80 ng) using Lipofectamine 2000 (0.2 µL) (Thermo Fisher Scientific) following the transient transfection method described in Section 4.5 and were cultured in 10% FBS-supplemented DMEM medium for 24 h. The culture medium was replaced with growth medium (100 µL) containing solvent (DMSO, 0.5%), and media supplemented with sesamol (1) or 4SCAP (14), or media without sesamol (1), were also added. After 2 h of incubation, the cell viability in these cultures was measured using a CellTiter-Glo^®^ luminescent cell viability assay kit.

### 4.9. Pro-Oxidant Activity of the Sesamol (1) Oxidation Product, Sesamol Oligomer

Using a method we previously reported [34], we prepared sesamol oligomers by oxidizing sesamol (1) (1 mM) and L-DOPA (0.1 mM) in 50 mM sodium phosphate buffer (pH 7.4) with tyrosinase (200 U) in 2 mL. The mixture was incubated at 37 °C for 120 min. Tyrosinase alone (100 U/mL) was used as a control. Subsequently, 2 mL of 2 mM GSH (1 molar equivalent to sesamol) was added to 2 mL oligomer solution and was then incubated at 37 °C. Starting from 0 min after adding GSH, 100 μL samples were taken at 30 and 60 min from the start of the reaction, mixed with 900 μL 0.4 M HClO_4_ to stop the oxidation reaction, after which GSH and GSSG were measured. Following the method described by Imai et al. [35], the reaction mixture was analyzed using an HPLC system with a 0.4 M HCOOH:MeOH mobile phase at 30:70 (*v*/*v*), a 294 nm UV detector, and a column temperature of 45 °C.

Simultaneously with the measurement of GSH and GSSG, H_2_O_2_ was analyzed by a spectrophotometric method according to Ito et al. [33]. For H_2_O_2_ measurement, the oxidation mixture was diluted 10-fold with pH 7.4 buffer, and these diluted mixtures were reacted with an equal amount of the chromogenic reagent Ampliflu™ Red to produce a red pigment with an absorption maximum at 568 nm [41]. The reaction mixture was allowed to stand at room temperature for 10 min, after which the absorption spectrum was promptly measured in the range of 450–650 nm. These procedures followed the manufacturer’s instructions (Invitrogen, Tokyo, Japan).

### 4.10. Statistical Analyses

Student’s *t*-tests (two-tailed) were performed using Microsoft Excel 2019, version 16.0 (Microsoft Co., Redmond, WA, USA). A *p*-value of <0.05 was considered statistically significant.

## 5. Conclusions

This study demonstrates that sesamol (1) embodies a significant toxicological paradox: while it is conventionally celebrated as a multi-functional systemic antioxidant and is recognized as a potent tyrosinase inhibitor, it undergoes tyrosinase-dependent metabolic bioactivation into a cytotoxic pro-oxidant specifically within melanin-producing cells. We successfully identified the reactive intermediate, sesamol-5,6-quinone (7), and its subsequent thiol adducts using hTYR-293T cells under sub-cytotoxic conditions, proving that sesamol (1) serves as an efficient substrate for bioactivation that initiates cellular damage through the arylation of intracellular protein thiols. Furthermore, the cytotoxicity of sesamol (1) is exacerbated by its oxidative metabolites. Sesamol oligomers exhibit potent pro-oxidant activity by facilitating a robust redox cycle that generates H_2_O_2_ and depletes reduced GSH through its oxidation to GSSG. A comparative analysis established a clear hierarchy of pro-oxidant potency among *p*-substituted phenolics: RD (3) > sesamol (1) > EQ (6) > RES (4)/PTS (5). These findings define the Systemic-Local Paradox: a multi-organ protector in most tissues is ‘hijacked’ by tyrosinase and transformed into a targeted toxin specifically within melanocytes. Consequently, the use of sesamol (1) and related phenolics in cosmetic and skin-whitening applications necessitates a careful re-evaluation of their safety profiles, as their biological impact is dictated by the specific metabolic landscape of the target cells. Finally, limitations of this study include (1) the lack of in vivo validation and (2) the absence of long-term exposure data. Future research should aim to determine the metabolic threshold for cytotoxic bioactivation to maximize the pharmacological benefits of these compounds while minimizing risks to skin health.

## Figures and Tables

**Figure 1:**
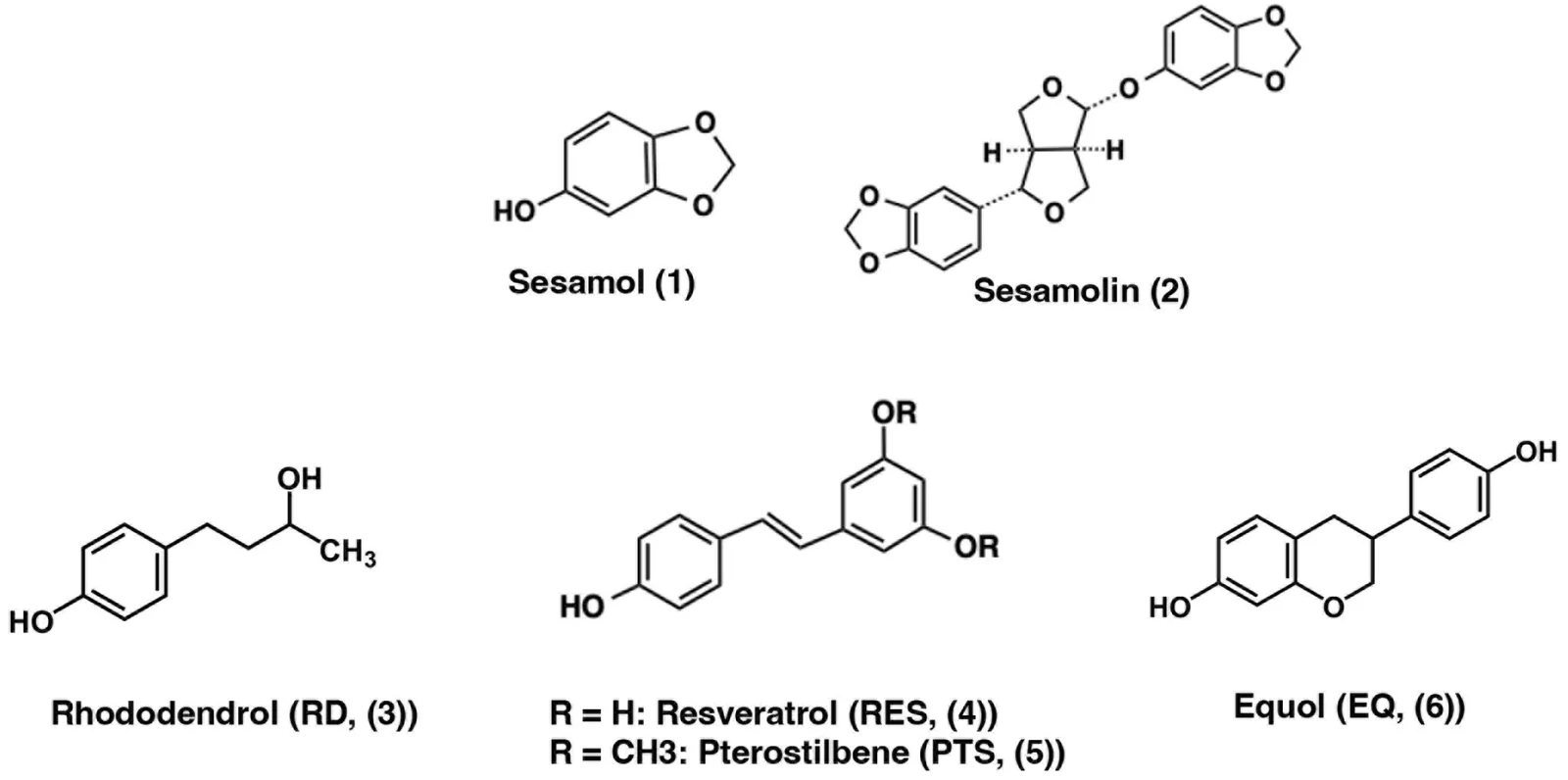
Chemical structures of sesamol (1), sesamolin (2), rhododendrol (RD) (3), resveratrol (RES) (4), pterostilbene (PTS) (5), and equol (EQ) (6).

**Figure 2:**
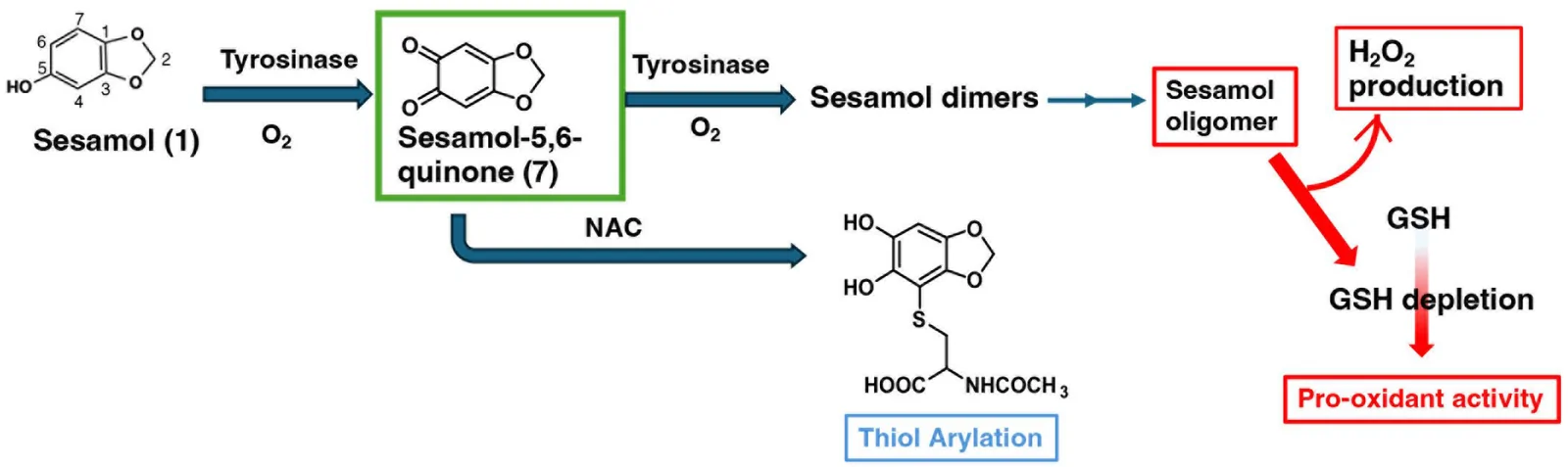
Proposed mechanism for sesamol (1)-induced pro-oxidant activity and thiol arylation via metabolic activation to sesamol-5,6-quinone (7).

**Figure 3:**
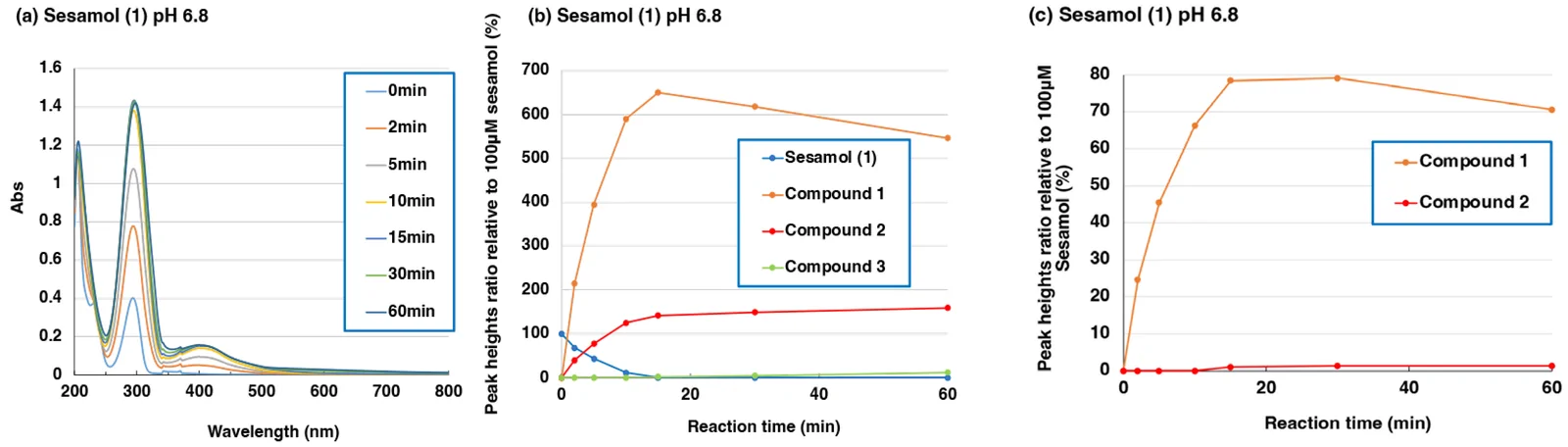
Reaction profiling and HPLC analysis of tyrosinase-mediated sesamol (1) oxidation. (**a**) Successive changes in the UV/visible absorption spectra of sesamol (1) monitored at pH 6.8. (**b**,**c**) Chromatographic detection at 298 nm (**b**) and 400 nm (**c**) of products following reaction termination with HClO_4_. Data represent consistent results obtained from duplicate experimental runs.

**Figure 4:**
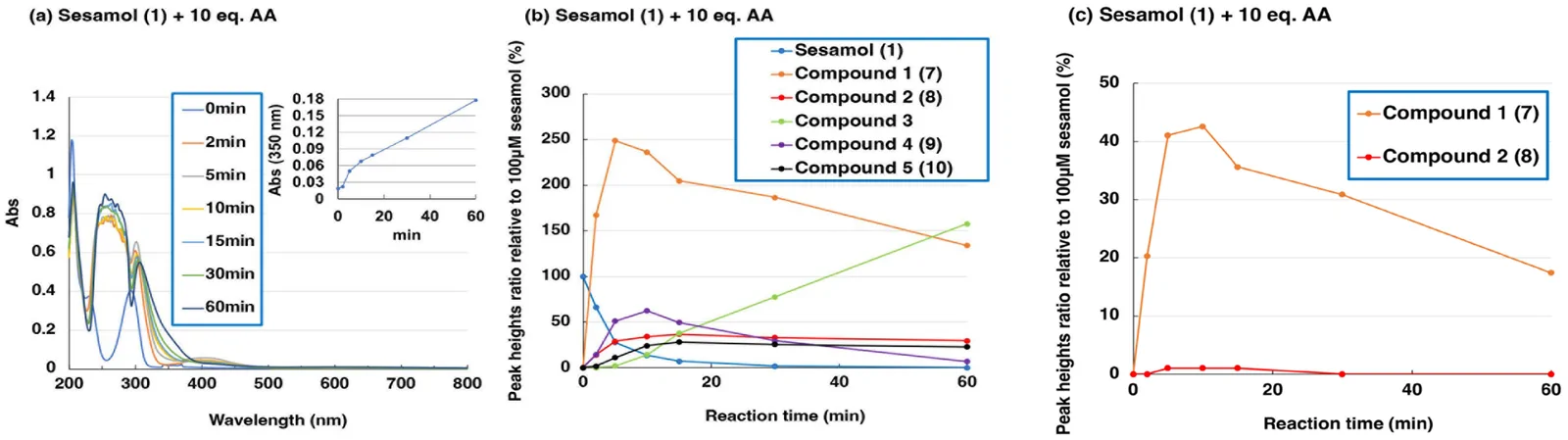
Time course of the tyrosinase-catalyzed oxidation of sesamol (1) in the presence of AA. (**a**) UV/visible spectral changes of sesamol (1) at pH 6.8 in the presence of 10 mol eq. AA. The inset shows the time-dependent change in absorbance at 350 nm. (**b**) HPLC analysis at 298 nm following the tyrosinase-catalyzed oxidation of sesamol (1) at pH 6.8 in the presence of 10 mol eq. AA, the reaction being stopped by HClO_4_. (**c**) HPLC analysis at 400 nm of the tyrosinase-catalyzed oxidation of sesamol (1). The experiments were performed in duplicate, and good reproducibility was obtained.

**Figure 5:**
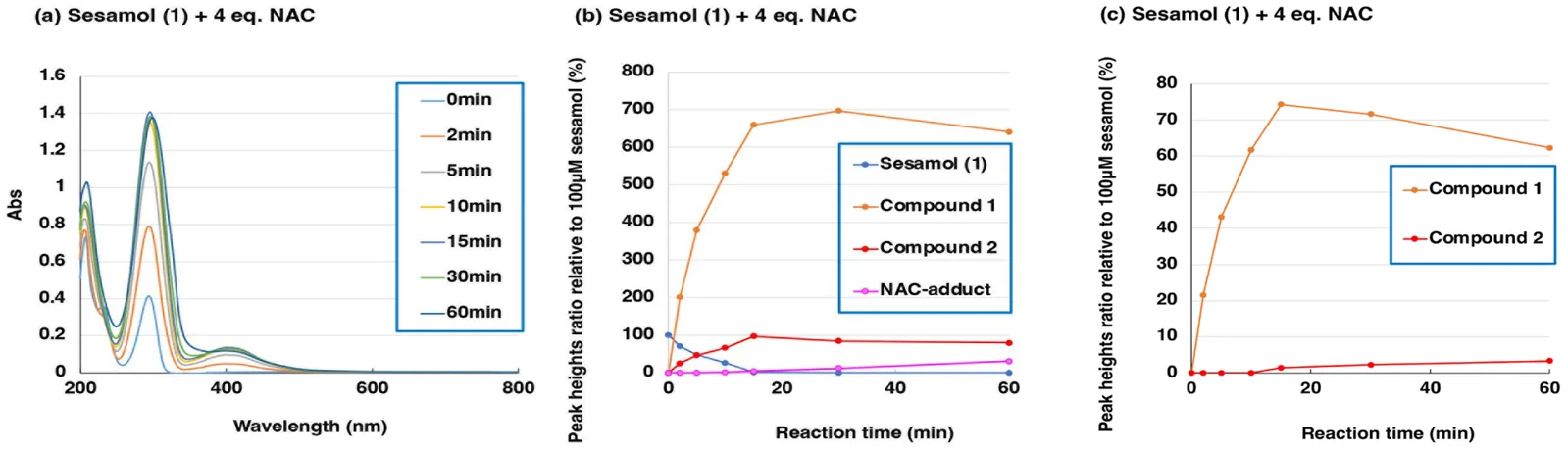
Reaction profiling and HPLC analysis of tyrosinase-mediated sesamol (1) oxidation in the presence of NAC. (**a**) Successive changes in the UV/visible absorption spectra of sesamol (1) at pH 6.8 supplemented with 4 molar equivalents of NAC. (**b**) HPLC analysis of the reaction at 298 nm following the tyrosinase-catalyzed oxidation of sesamol (1), with the reaction being quenched with HClO_4_. (**c**) HPLC analysis at 400 nm of the tyrosinase-catalyzed oxidation of sesamol (1). The data are averages from two independent experiments with good reproducibility.

**Figure 6:**
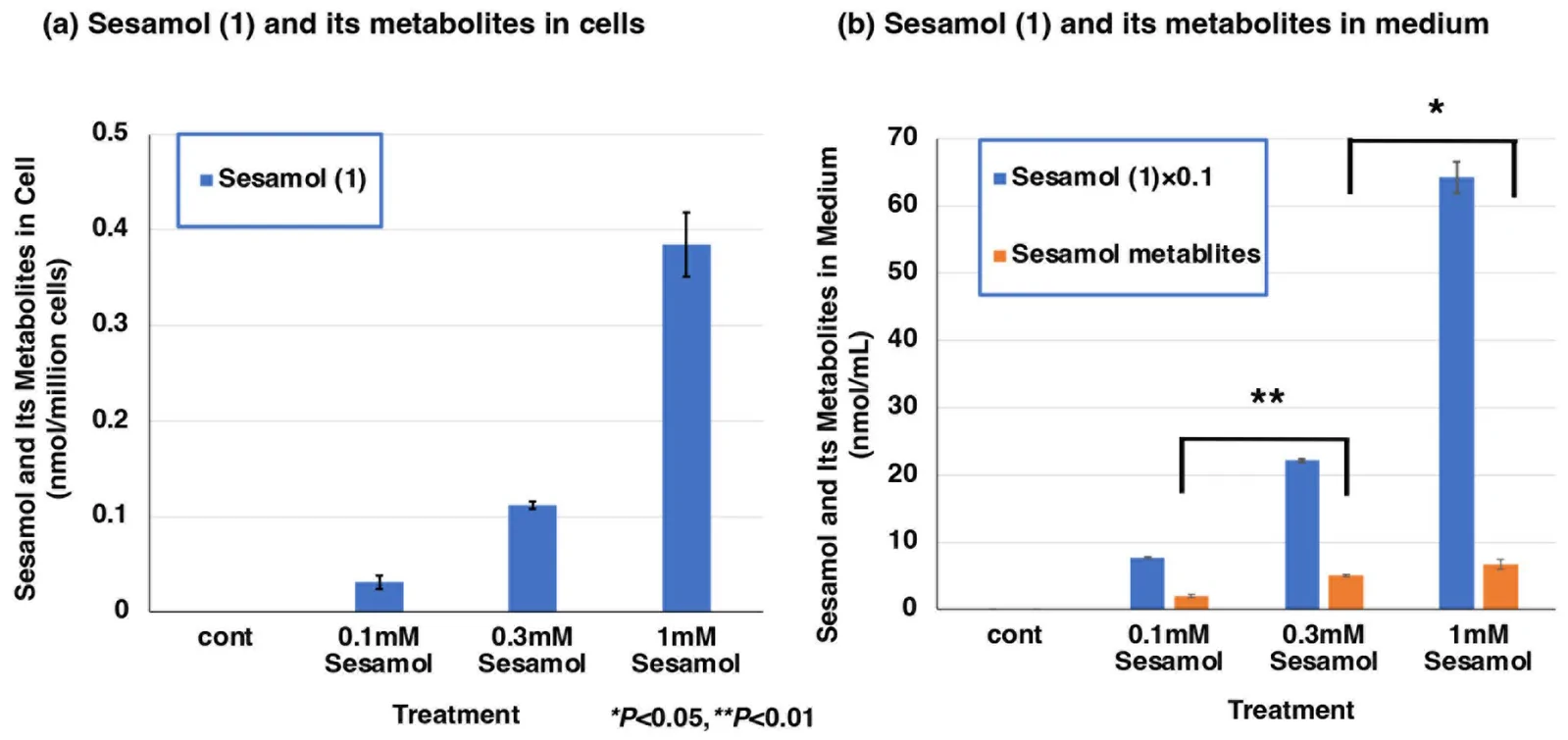
Identification of CySH and GSH adducts generated via sesamol (1) metabolism in hTYR-293T cells. (**a**) Intracellular levels of sesamol (1) and its metabolic derivatives. (**b**) Concentration of sesamol (1) measured in the culture medium. Notably, the levels of metabolic products within the cells remained below the analytical detection limit. Results are presented as mean ± standard deviation (*n* = 3). Significant differences between treatment groups are indicated by asterisks (* *p* < 0.05, ** *p* < 0.01), with statistical analysis performed using a two-tailed Student’s *t*-test.

**Figure 7:**
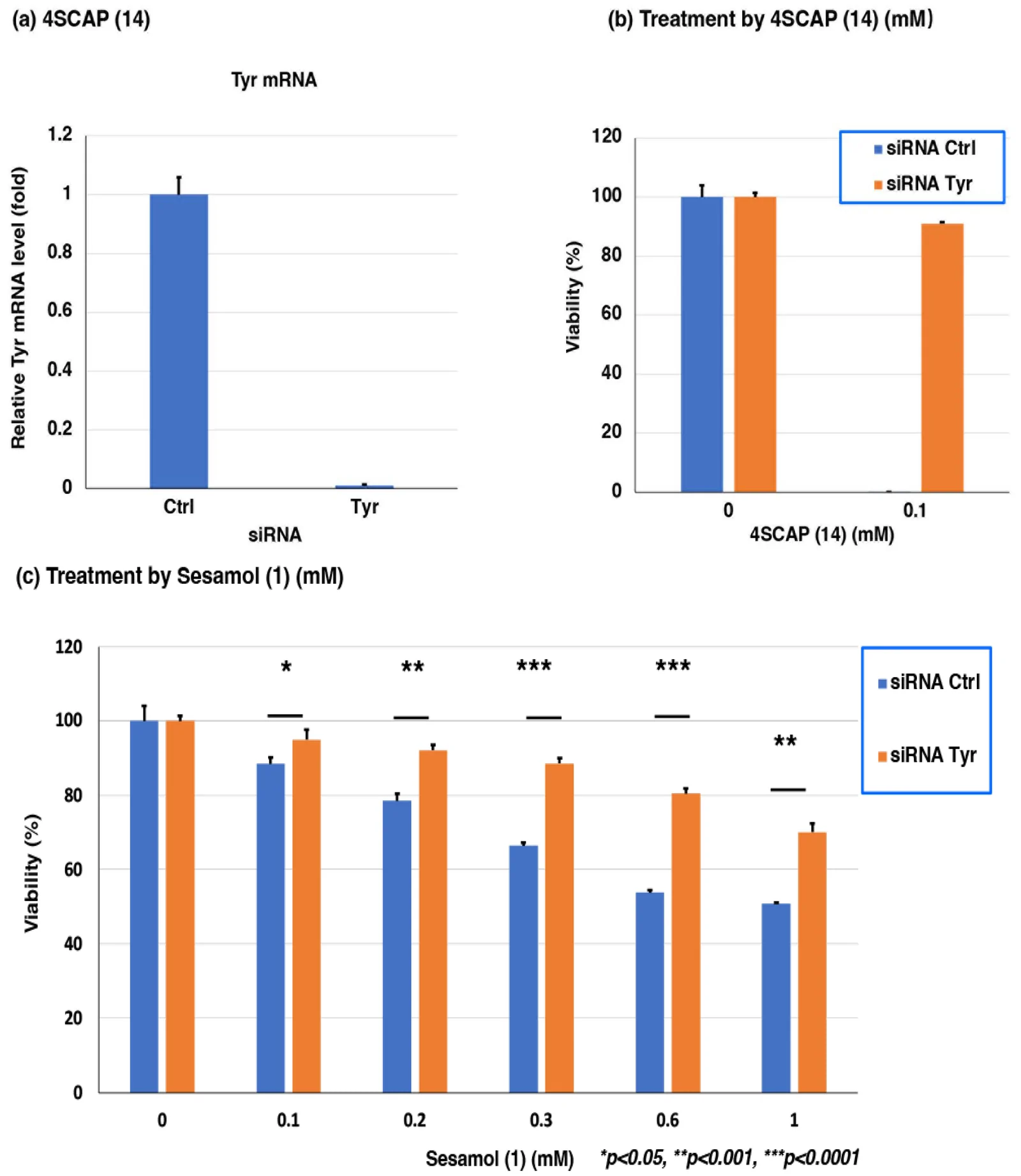
Tyrosinase-mediated cytotoxicity and the effect of gene silencing in B16BL6 melanoma cells. (**a**) Suppression of Tyr mRNA levels in cells following siRNA transfection. (**b**,**c**) Dose-dependent reduction in viability by 4SCAP (14, (**b**)) and sesamol (1, (**c**)), showing dependence on tyrosinase activity. Following a 24 h transfection with either control or Tyr-targeted siRNA, cells were exposed to the indicated compound concentrations for an additional 24 h. Survival rates and mRNA expression (in vehicle groups) were then determined. Data are presented as mean ± SD (*n* = 3) and are representative of two independent trials. Asterisks (* *p* < 0.05, ** *p* < 0.001, *** *p* < 0.0001) denote statistical significance compared to the control at each concentration, evaluated using a two-tailed Student’s *t*-test.

**Figure 8:**
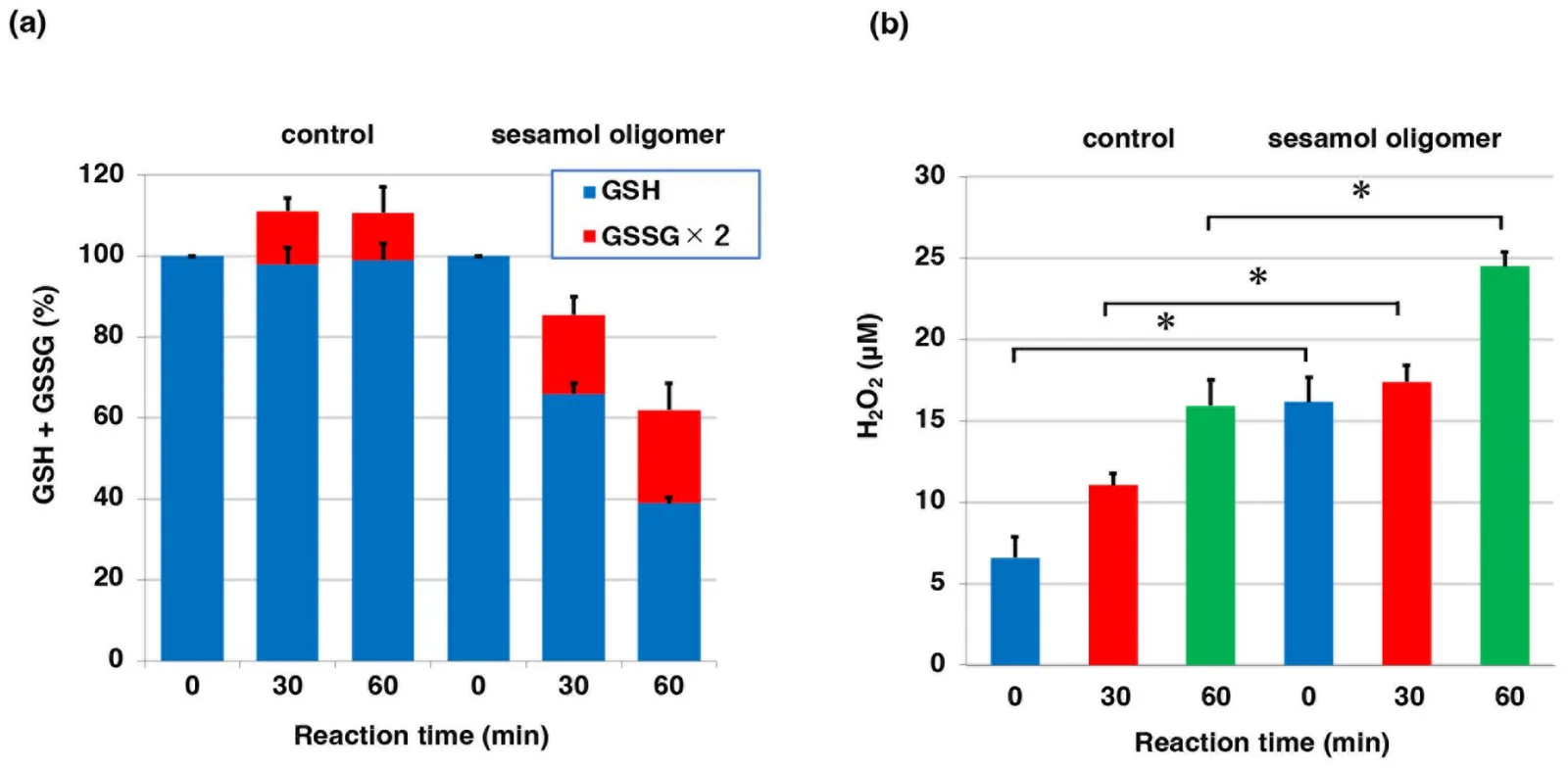
Pro-oxidant effects of sesamol oligomers: GSH depletion and H_2_O_2_ generation. (**a**) Levels of GSH oxidation and the resulting formation of GSSG starting with 1 molar equivalent of GSH. (**b**) Time-dependent production of H_2_O_2_ measured at baseline and after 30 and 60 min of incubation. Values are presented as the mean ± standard error (SE) of three independent trials. Asterisks (* *p* < 0.01) denote statistical significance.

**Table 1:** Comparison of pro-oxidant potency.

Compound (Oligomer)	GSH Depletion (at 60 min)	H_2_O_2_ Generation (at 60 min)	Relative Potency & Key Features
Sesamol (1)	ca. 60%	25 µM	Highly Potent: exhibits significantly higher H_2_O_2_ production compared to RES (4) and EQ (6)
Rhododendrol (RD, (3))	ca. 90%	26 µM (at 30 min)	Most Potent: extremely high capacity for GSH depletion and ROS generation
Resveratrol (RES, (4))	ca. 50%	Negligible (control level)	Relative Weak: high GSH reduction but lacks significant H_2_O_2_ generation
Pterostilbene (PTS, (5))	(similar to RES (4))	Negligible	Relative Weak: exhibits a profile consistent with its parent compound RES (4)
Equol (EQ, (6))	ca. 60%	21 µM	Highly Potent: shows similar GSH depletion to sesamol (1) but lower H_2_O_2_ generation

Summary of Findings: The pro-oxidant activities of compounds (3), (4), (5), and (6) were evaluated under the same conditions as those used for sesamol (1). Potency Hierarchy: Our comparative data establish a clear hierarchy of pro-oxidant activity: RD (3) > Sesamol (1) > EQ (6) > RES (4)/PTS (5). Sesamol’s Distinct Profile: While sesamol (1) and EQ (6) show similar levels of GSH depletion (ca. 60%), sesamol oligomers generate a higher concentration of H_2_O_2_ (25 µM) compared to EQ (6) (21 µM). This underscores the robust capacity of sesamol (1) to facilitate redox cycling. Contrast with RES (4)/PTS (5): Unlike RES (4) and PTS (5), which produce negligible H_2_O_2_ above control levels, sesamol (1) acts as a much more aggressive pro-oxidant. Toxicological Implication: These findings highlight that the tyrosinase-mediated bioactivation of sesamol (1) not only results in direct protein arylation but also induces a profound redox imbalance through indirect oxidative stress. This dual mechanism drives its selective cytotoxicity toward melanin-producing cells, defining the Systemic-Local Paradox.

## Data Availability

The original contributions presented in this study are included in the article/Supplementary Material. Further inquiries can be directed to the corresponding author.

## Supplementary Materials

The following supporting information can be downloaded at: https://www.mdpi.com/article/10.3390/ijms27198597/s1.

## Abbreviations

The following abbreviations are used in this manuscript:

AA	L-Ascorbic acid
4SCAP	4-*S*-cysteaminylphenol
Ampliflu^™^ Red reagent	10-Acetyl-3,7-dihydroxyphenoxazine
ATCC	American Type Culture Collection
CySH	Cysteine
DBBQ	3,5-di-*tert*-butyl-1,2-benzoquinone
DMEM	Dulbecco’s modified Eagle’s medium
DMSO	Dimethyl sulfoxide
EQ	Equol (7-hydroxy-3-(4-hydroxyphenyl)-chroman)
FBS	Fetal bovine serum
GSH	Glutathione
GSSG	Oxidized glutathione
HClO_4_	Perchloric acid
HPLC	High-performance liquid chromatography
hTYR-293T	Human tyrosinase-expressing 293T
L-DOPA	(3,4-Dihydroxyphenyl)-L-alanine
MC1R	Melanocortin 1 receptor
MITF	Microphthalmia-associated transcription factor
NaBH_4_	Sodium borohydride
NAC	*N*-Acetyl-L-cysteine
NMR	Nuclear magnetic resonance
Nrf2	Nuclear factor-erythroid 2-related factor 2
PTS	Pterostilbene
RD	Rhododendrol (4-(4-hydroxyphenyl)-2-butanol)
RES	Resveratrol
ROS	Reactive oxygen species
RT	Retention times
siRNA	Small interfering RNA
TRP-1	Tyrosinase-related protein-1
TRP-2	Tyrosinase-related protein-2
UV	Ultraviolet
