# Supplementary material for: The Dual Nature of Sesamol: Tyrosinase-Dependent Bioactivation from an Inhibitor to a Cytotoxic Pro-Oxidant
Source: Int J Mol Sci. 2026 Sep 25;27(19):8597. doi: 10.3390/ijms27198597 (PMC13665154; doi:10.3390/ijms27198597)
Supplement: Supplementary file 1 [file ijms-27-08597-s001.zip › ijms-4485186-supplementary.pdf]

Figure S1. Representative HPLC chromatogram of sesamol oxidation in the presence of ascorbic acid. HPLC chromatogram of the tyrosinase-catalyzed oxidation of 100 μM sesamol (1) for 30 min in the presence of 1000 μM AA (10 molar eq.) at pH 6.8. #1: Compound 3 (RT 4.5 min), #2: Compound 5 (RT 5.5 min), #3: Compound 1 (RT 5.9 min), #4: Compound 2 (RT 7.6 min), and #5: Compound 4 (RT 11.7 min).

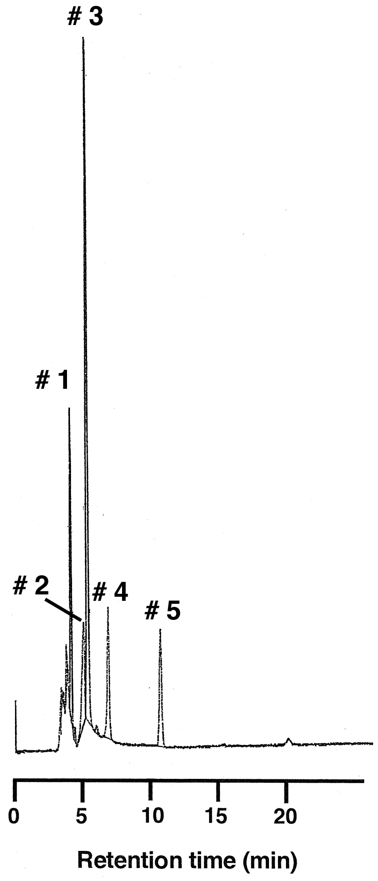

Figure S2: ^{1}H-NMR of 5,6-diacetoxy-1,3-benzodioxole (11) (CD_3OD)

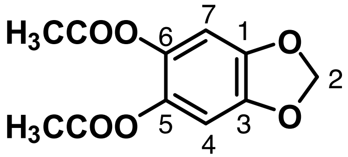

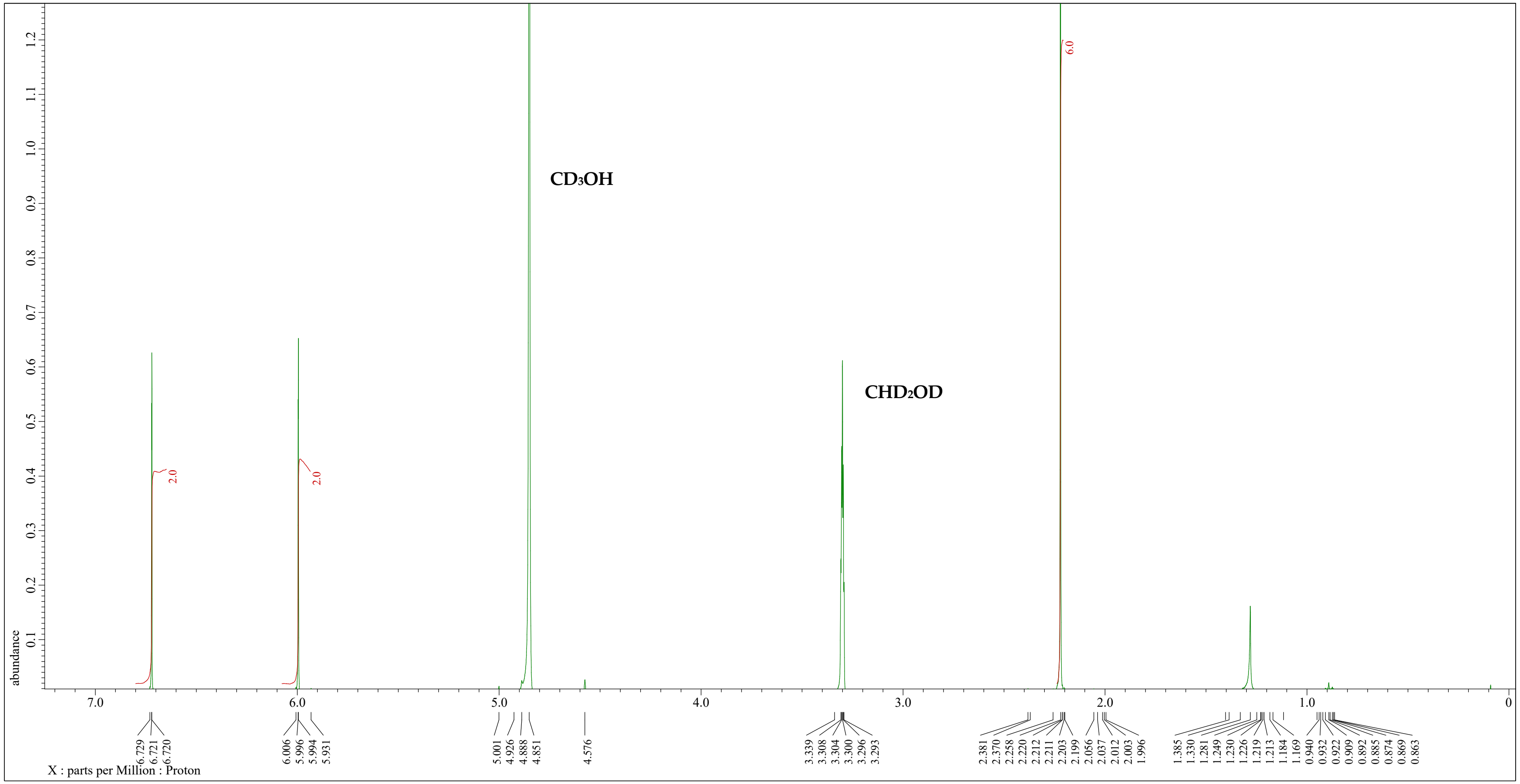

Figure S3: ^{1}H-NMR: 4,5-diacetoxy-1,3-benzodioxole (12) ($CDCl_3$)

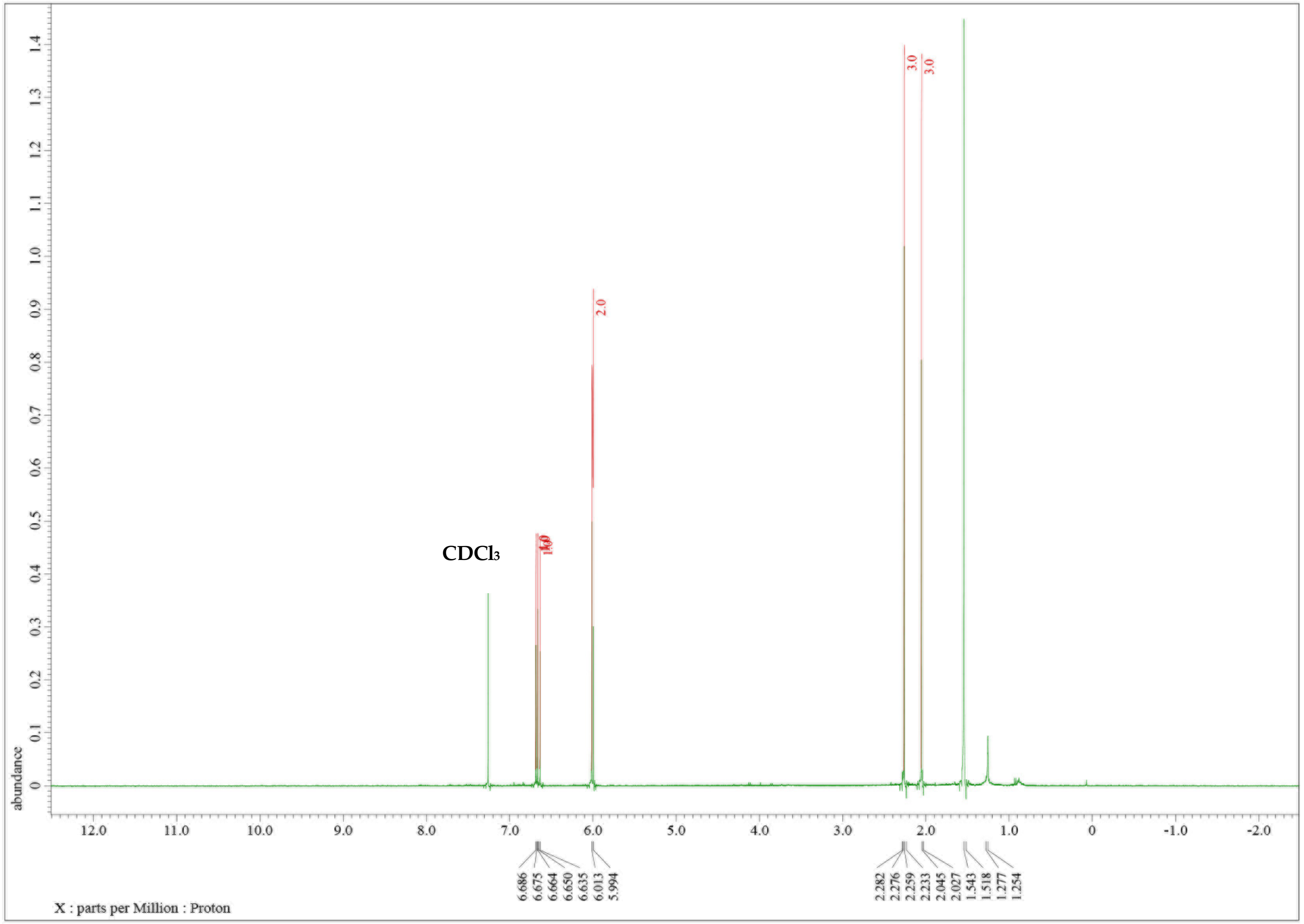

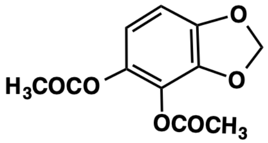

Figure S4: ^{1}H-NMR: 4-NAC-5,6-dihydroxy-1,3-benzodioxole (13) ($CDCl_3$)

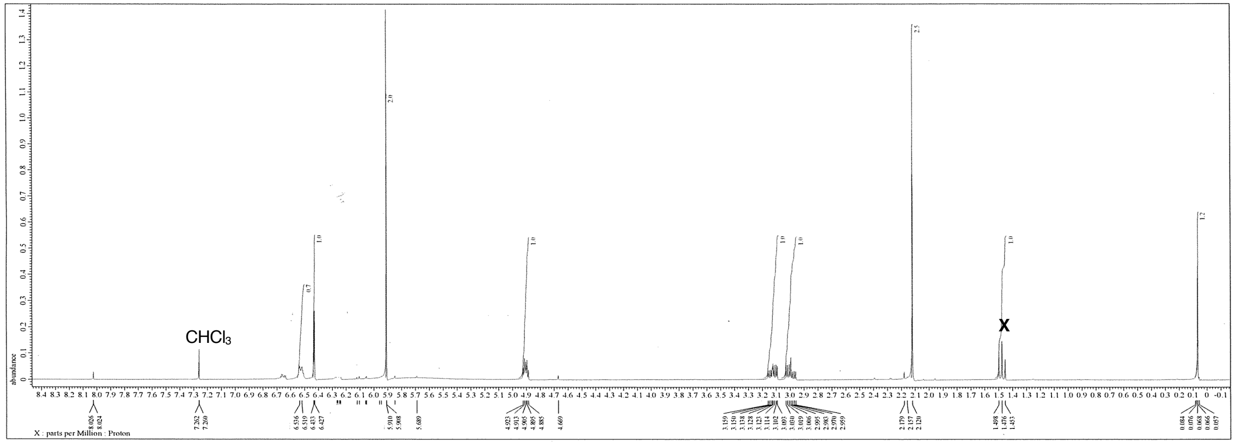

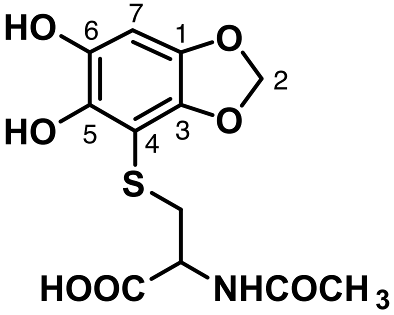

X is a peak derived from the contaminating impurity.

Figure S5: ^{13}C-NMR: 4-NAC-5,6-dihydroxy-1,3-benzodioxole (13) ($CDCl_3$)

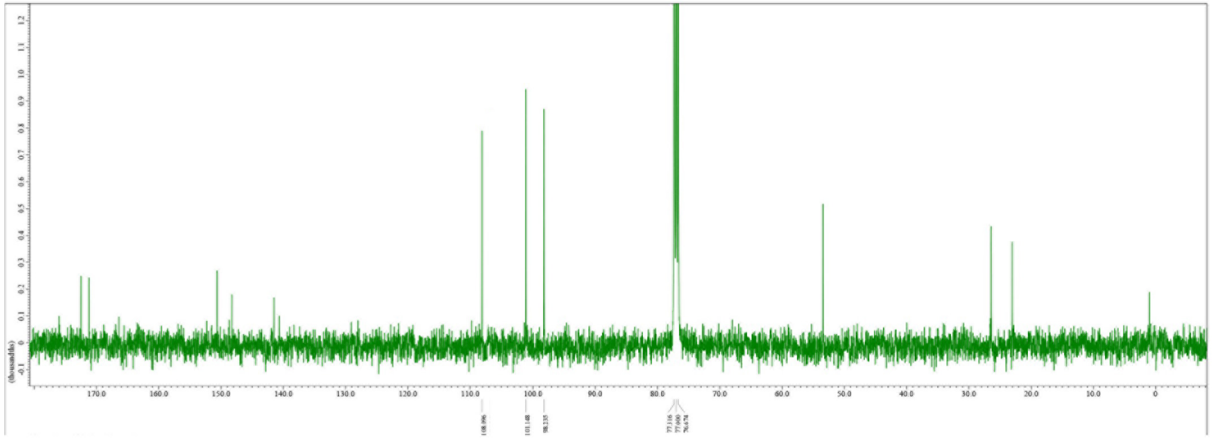

Figure S6. In human tyrosinase-expressing hTYR-293T cells, tyrosinase-dependent cytotoxicity against (a) Sesamol (1), and (b) 4SCAP (14). Cells transfected with TYR- or Mock- were treated with the indicated concentrations of the compound for 2 h, and their viability was evaluated. Data represent means ± SD (n = 3 wells). Results are representative of two independent experiments. Statistically significant differences: *$p < 0.005$.

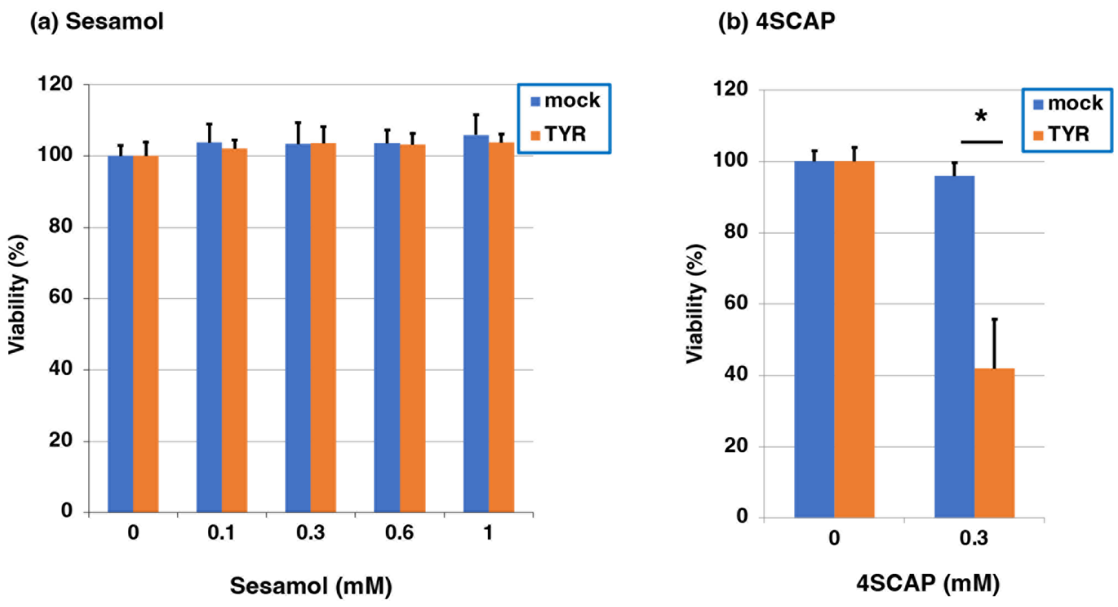

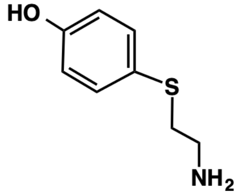

4SCAP (14)

Table S1. Correspondence table of some compounds detected in the HPLC chromatogram

HPLC Compound 1–6 correspondence					
**HPLC label**	**Retention Time (min)**	**Structure No.**	**Current assignment**	**Status / note**	**Structure**
**Compound 1**	≈5.9	(7)	Sesamol-5,6-quinone	Tentatively assigned as the major 5,6-quinone product; later identified as sesamol-5,6-quinone (7).	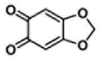
**Compound 2**	≈7.6	(8)	Sesamol-4,5-quinone	Tentatively assigned as the positional isomer, sesamol-4,5-quinone (8).	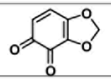
**Compound 3**	≈4.5	—	Secondary coupling product / possible dimeric species	Assumed to be a dimer/coupling product based on its time-dependent increase and enhanced formation in the presence of AA; structure not established.	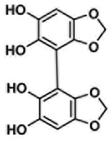
**Compound 4**	≈11.7	(9)	Sesamol-5,6-catechol / 5,6-dihydroxy-1,3-benzodioxole	Initially tentatively assigned as a catecholic product; subsequently identified as 5,6-dihydroxy-1,3-benzodioxole (9) using the stable diacetyl derivative (11) for NMR characterization.	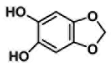
**Compound 5**	≈5.5	(10)	Sesamol-4,5-catechol / 4,5-dihydroxy-1,3-benzodioxole	Tentatively assigned as sesamol-4,5-catechol (10); positional-isomer assignment supported by the 4,5-diacetoxy derivative (12).	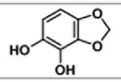
**Compound 6**	≈8.4	(13)	4-NAC-5,6-dihydroxy-1,3-benzodioxole	Structural assignment of Compound 6 as the mono-NAC derivative (13), based on isolation after $NaBH_4$ treatment and $^{1}H/^{13}C$-NMR characterization.	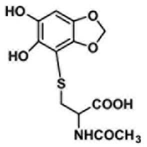

Table S2. Assignment of ^{1}H and ^{13}C of Sesamol metabolites (11), (12), and (13)

Position	5,6-diacetoxy-1,3-benzodioxole (11)	4,5-diacetoxy-1,3-benzodioxole (12)	4-NAC-5,6-dihydroxy-1,3-benzodioxole (13)	Position	4-NAC-5,6-dihydroxy-1,3-benzodioxole (13)
H-2	5.99 (s, 2H)	6.00 (d, 2H, J =7.6 Hz)	5.89 (s, 2H)	C-1	150.5
H-4	6.72 (s, 1H)			C-2	101.2
H-6		6.65 (d, 1H, J = 10.2 Hz)		C-3	141.5
H-7	6.72 (s, 1H)	6.67 (d, 1H, J = 10.2 Hz)	6.41 (s, 1H)	C-4	98.2
$SC\underline{H}_2CH$			2.99 (m, 1H), 3.10 (m. 1H)	C-5	148.5
$SCH_2C\underline{H}$			4.89 (m, 1H)	C-6	140.5
$COC\underline{H}_3$	2.21 (s, 6H)	2.03 (s, 3H), 2.26 (s, 3H)	2.14 (s, 3H)	C-7	108.1
				$SCH_2\underline{C}H$	53.5
				$S\underline{C}H_2CH$	27.0
				$\underline{C}OCH_3$	172.5
				$CO\underline{C}H_3$	23.0
				$\underline{C}OOH$	171.0

The spectra of compounds (11) were taken in CD_3OD, and (12) and (13) were taken in $CDCl_3$. Chemical shifts are shown in ppm relative to the solvent signal at 3.30 ppm (CD_3OD) or 7.26 ppm ($CDCl_3$) in ^{1}H-NMR, and 77.0 ppm in ^{13}C-NMR, respectively.
